# Coverage, Traits, and Geographic Distribution of Online Surgeon Reviews: Large-Scale Cross-Sectional Analysis

**DOI:** 10.2196/79427

**Published:** 2026-04-14

**Authors:** Michael Geng, Carlos Riveros, Yash B Shah, Sanjana Ranganathan, Kai Fok, Renil Sinu Titus, Vatsala Mundra, Eusebio Luna Velasquez, Dharam Kaushik, Allan S Detsky, Angela Jerath, Benjamin N Breyer, Yusuke Tsugawa, Christopher J D Wallis, Raj Satkunasivam

**Affiliations:** 1 Texas A&M Health Science Center, Houston, TX, United States; 2 Department of Urology, Houston Methodist Hospital, 6560 Fannin Street, Suite 2100, Houston, TX, 77030, United States, 1 (713) 441-6455, 1 (713) 441-6463; 3 Thomas Jefferson University, Philadelphia, PA, United States; 4 University of Toronto, Toronto, ON, Canada; 5 Sunnybrook Health Science Centre, Toronto, ON, Canada; 6 University of California, San Francisco, San Francisco, CA, United States; 7 University of California, Los Angeles, Los Angeles, CA, United States

**Keywords:** online ratings, surgeons, demographics, coverage, reviews

## Abstract

**Background:**

The use of online physician rating platforms has significantly increased and has been shown to influence physician selection. There are limited data on the use of these platforms for rating surgeons.

**Objective:**

In this study, we sought to assess the geographic distribution of and patterns in rating scores of surgeons in the United States. Additionally, we examined rating volumes across different surgical specialties and the association between peer-nominated and patient-initiated ratings on online rating platforms in the United States.

**Methods:**

We conducted a cross-sectional study by identifying 201,154 surgeons in the United States via the National Plan and Provider Enumeration System records and Doctors and Clinicians downloadable file. We assessed surgeon coverage on 3 online rating platforms and their geographic use patterns. We described the rating scores and volumes across different surgical specialties and assessed the relationship between rating platforms by comparing peer-nominated and patient-initiated online ratings.

**Results:**

A total of 78.86% (158,630/201,154) of the surgeons had ratings on at least 1 of the 3 patient-initiated websites across 11 specialties. Plastic surgeons, neurosurgeons, and orthopedic surgeons had the highest mean number of patient-initiated ratings. Surgeons with “Top Doctor” recognition from peers (23,171/201,154, 11.52%) were associated with an increased median patient-initiated rating (Healthgrades: 4.36, IQR 3.88-4.71 vs 4.20, IQR 3.64-4.64, *P*<.001, and *r*=0.09; Vitals: 4.30, IQR 4.00-4.60 vs 4.20, IQR 3.80-4.50, *P*<.001, and *r*=0.09; RateMDs: 4.20, IQR 3.80-4.50 vs 3.80, IQR 3.60-4.60, *P*<.001, and *r*=0.16). Geographic analysis indicated that 91.06% (295,816,471/324,870,510) of the US population lives in a county with a surgeon rated 10 times or more.

**Conclusions:**

Both patient-initiated and peer-nominated rating platforms have a comprehensive coverage of surgeons in the United States, but this coverage differs significantly between surgical specialties. Further work should assess how publicly available online ratings drive surgeon selection and their association with patient experience and postoperative outcomes.

## Introduction

The use of online physician rating platforms is steadily increasing and plays a critical role in patients’ selection of health care providers. According to recent surveys, 86% of respondents reported reading online reviews before choosing a physician, and 73% indicated a preference for physicians with ratings of 4 out of 5 or higher [1,2] These figures underscore the substantial influence of such platforms (in addition to other factors such as insurance coverage and referral patterns) on patients’ choices.

These rating platforms encompass both peer-nominated ratings, which include physician-nominated lists that designate high-performing physicians as “Top Doctors,” and patient-initiated ratings that collate patient-submitted reviews and ratings of their experiences with physicians. These platforms offer potential and current patients a wealth of information, including narrative reviews; numerical ratings for specific dimensions; and an overall rating of care, typically using a 5-point scale. The influence of online ratings may also extend to clinic volumes, with some surgeons raising concerns regarding the possible disproportionate use of these open-forum platforms to share complaints [3,4]. To evaluate whether online ratings reflect the quality of care delivered, prior work has reported no association between ratings and objective quality metrics or peer assessments of clinical performance [5]. Health care institutions have likewise become increasingly attentive to their online reputations with an increasing number of patients using these platforms [6,7].

In studies focusing specifically on surgical practices, factors associated with more favorable online ratings have included active X (formerly known as Twitter) use and shorter patient-reported clinic wait times among orthopedic surgeons [8], lower clinic volumes among urologists in California [9], and qualitative attributes (such as perceived trustworthiness, punctuality, and ease of scheduling) as strong predictors of higher ratings [10]. Disparities across geographic regions (eg, underserved or rural areas) and across surgical specialties with differing practice styles (such as variations in clinical acuity and patient volume) may further influence the likelihood of appearing on curated lists or receiving favorable ratings. However, the scope of prior investigations has been limited by the number of specialties examined and the geographic coverage of surgeons included on both patient-initiated and peer-nominated platforms.

To our knowledge, a large-scale study assessing the representation of the entire cohort of surgeons in the United States on physician rating platforms has not been performed. By creating a national surgeon directory from datasets published by the US Centers for Medicare and Medicaid Services (CMS), the country’s largest health care payer, we sought to address this gap in the literature by quantifying surgeon representation on the predominant online rating platforms nationally, describing any geographic differences in online representation, comparing the representation of surgeons between patient-initiated and peer-nominated physician rating platforms, and assessing specialty-specific differences in surgeon representation among these platforms.

Our research questions are as follows:

1. What is the overall coverage of surgeons on the 3 major online physician rating platforms in the United States?
2. Are there county-level regional differences in surgeon representation on online rating platforms in the United States?
3. What is the relationship between overall rating and rating volume on patient-initiated rating platforms and peer-nominated “Top Doctor” status among surgeons in the United States?
4. How does surgeon visibility on these online patient-initiated and peer-nominated rating platforms differ across different subspecialties in the United States?
5. Are there differences in the demographics of highly reviewed surgeons?

## Methods

This study followed the STROBE (Strengthening the Reporting of Observational Studies in Epidemiology) reporting guidelines [11].

### Ethical Considerations

The data used to conduct this study are publicly available. No patient data were involved. The study was exempt from institutional review board approval and requirement for informed consent. Research data, including individual surgeon-level information, were deidentified. No compensation was provided for the publicly available data of the included surgeons.

### Identification of Patient-Initiated and Peer-Nominated Physician Rating Platforms

We identified the most popular patient-initiated and peer-nominated rating platforms based on search traffic (via Semrush, which tracks the reach of a website by looking at the number of its backlinks) [12]. We identified 3 patient-initiated rating platforms (RateMDs, Vitals, and Healthgrades) and 1 peer-nominated “Top Doctor” rating platform (Castle Connolly [13]) that had the highest number of backlinks on Semrush and had the largest volume of recognized surgeons nationally. Castle Connolly is a private consumer research firm that distinguishes the nation’s top 7% of physicians through a physician peer nomination process and awards “Top Doctor” status based on these nominations. These lists are prepared for each specialty and most subspecialties. As the largest physician-nominated “Top Doctor” publication, prior studies have used Castle Connolly to assess the validity and role of physician-nominated ratings [14].

### Cohort Derivation: Surgeon Identification and Linkage to Patient-Initiated and Peer-Nominated Ratings

**Figure 1.**
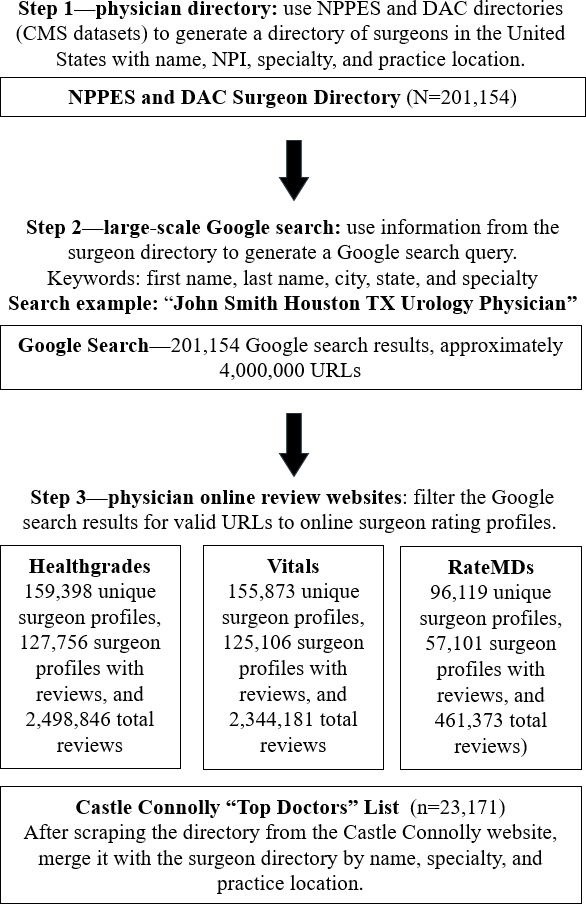
Methodology workflow. We created a surgeon directory using the National Plan and Provider Enumeration System (NPPES) and Doctors and Clinicians (DAC) surgeon directories from the Centers for Medicare and Medicaid Services (CMS). Next, we performed automated Google searches for each physician to find the rating platforms of interest. We then extracted the ratings from each rating website of interest and merged them with the “Top Doctors” list. NPI: National Provider Identifier.

We began by identifying all currently practicing surgeons in the United States using 2 publicly available datasets maintained by the US CMS (step 1 of Figure 1). The National Plan and Provider Enumeration System is a mandatory national registry of licensed health care providers and includes a unique National Provider Identifier, self-reported specialty, and practice location [15]. To ensure active clinical practice and improve specialty verification, we linked National Plan and Provider Enumeration System records to the CMS Doctors and Clinicians downloadable file, which contains physicians actively enrolled in and billing Medicare services [16]. Together, these datasets enabled comprehensive nationwide identification and subspecialty stratification of practicing surgeons by providing us with their registered National Provider Identifier, name, primary practice location, and specialty.

### Extraction of Data

We performed web scraping by adapting Python scripts from the GitHub repository linked by Wang et al [17]. From our surgeon directory, we programmatically ran Google searches using name, location, and specialty (step 2 of Figure 1). Through recording the top Google search results, we could reliably find a website link to each surgeon’s online patient-initiated rating profiles (Healthgrades, Vitals, and RateMDs). With a website link to each patient-initiated rating profile, we could then extract the total number of ratings and overall ratings on each surgeon profile (step 3 of Figure 1). An additional script was written to web scrape the peer-nominated rating platform (August 2022) and create a directory of listed physicians who were also recognized by Castle Connolly as “Top Doctors.” Web scraping of the patient-initiated physician rating platforms was performed between November 2022 and January 2023. Subsequent data cleaning was performed in the Python programming language (version 3.9; Python Software Foundation) and R (version 4.2.2; R Foundation for Statistical Computing).

### Outcomes

Descriptive statistics regarding the number of surgeons on at least one of these rating platforms; median number of ratings; and the proportions with at least one rating, 10 or more ratings, 25 or more ratings, 50 or more ratings, and 100 or more ratings stratified by specialty were reported. The number of ratings required to meet the top 1, 5, 10, 25, and 50 percentiles of surgeons across different specialties on patient-initiated rating platforms was reported. We reported the number of surgeons, stratified by specialty, who were given ratings of 4, 4 to 4.5, and 4.5 or above out of 5. We analyzed the use of different rating platforms by comparing the number of ratings across surgical specialties. To compare surgeon ratings, we computed a weighted average to create an aggregate rating across all 3 platforms by multiplying each platform’s average rating by its number of reviews, summing these values, and dividing by the total number of reviews. Comparing a weighted average allowed us to normalize for differing rating volumes across platforms.

To ensure adequate review sampling, we restricted visualization to surgeons with 10 patient-initiated ratings or more (the median number of ratings) and mapped their geographic distribution. Mapping of the geographic distribution of surgeons with 10 or more ratings and “Top Doctor” status across counties (defined as the primary administrative division of a state in the United States) [18] in the continental United States was performed. The top 25 counties with the highest number of surgeons rated as “Top Doctors” and surgeons with 10 or more ratings were reported.

Comparisons of ratings across the 3 patient-initiated platforms were made by stratifying by “Top Doctor” status to understand the association between physician- and patient-initiated ratings.

### Statistical Analysis

We analyzed the number of ratings and rating scores across specialties. Surgeon ratings were categorized into a 3-level classification based on prior research: low (<4.0), medium (4.0‐4.49), and high (≥4.5) [19]. A weighted average was calculated by adding the product of the number of ratings and average rating on each platform and then dividing by the total number of ratings across all platforms. This calculation was done to account for a surgeon’s variation in number of ratings across each patient-initiated rating platform. Geographic analysis was conducted to determine the geographic distribution of surgeons with patient-initiated ratings and “Top Doctor” designation. Specifically, we normalized the surgeon population using percentages of the total number of surgeons within a county. We linked zip codes to Federal Information Processing Standard codes that corresponded to a county. We explored the relationship between “Top Doctor” designation and patient-initiated ratings. We observed a left skew of the rating scores with clustering near the upper bound of the scale consistent with known ceiling effects in online physician ratings. We also observed a right skew of the number of ratings due to the large ranges encountered in the number of ratings across surgeon online profiles. Normality was assessed by plotting histograms for overall rating and number of ratings. We used nonparametric statistical tests as a result, and more specifically, we conducted comparisons between “Top Doctor” surgeons and non–“Top Doctor” surgeons using the Mann-Whitney *U* test. Across the 3 patient-initiated online rating platforms, the rating scores and number of ratings were compared after being stratified by “Top Doctor” status. Rank biserial correlation was used as the nonparametric metric of effect size for each Mann-Whitney comparison. It was derived by dividing the statistic from the Mann-Whitney *U* test by the square root of the sample size. Finally, we identified surgeons with the highest number of ratings (top 1%) to define the characteristics of this unique surgeon population. Data analysis and visualization were performed using R (version 4.2.2).

## Results

### Characteristics of Surgeons on Online Rating Platforms

Of the 201,154 unique surgeons identified, 189,463 (94.19%) had a profile on a patient-initiated rating platform, and 158,630 (78.86%) had at least one rating on a platform (Figure 1). Plastic surgery, colorectal surgery, and orthopedic surgery had the highest percentage of surgeons with at least one rating at 85.46% (6038/7065), 84.57% (1683/1990), and 84.01% (28,237/33,611), respectively (Table 1). General surgery and cardiothoracic surgery had the lowest percentage of surgeons with at least one rating at 67.96% (26,738/39,343) and 74.77% (4116/5505), respectively (Table 1). Among all surgeons with at least one rating, the mean and median number of ratings were 26.37 (SD 53.24) and 10 (IQR 1-32), respectively. Using the weighted average of the ratings across all 3 patient-initiated rating platforms, we found that the mean rating was 4.12 (SD 0.78) out of 5 and the median was 4.26 (IQR 3.71-4.70) out of 5 (Table S1 in Multimedia Appendix 1).

**Table 1.** Descriptive information of surgeons in the United States across specialties on peer-nominated rating website recognition along with specialty-specific counts of total review thresholds (reviews aggregated across all patient-initiated rating platforms) in this cross-sectional study (N=201,154).

Specialty	“Top Doctor” designation, n (%)	Rating count, median (IQR)	Rating count, mean (SD)	Surgeons with an online profile on ≥1 patient-initiated review platform, n (%)	Surgeons with ≥1 total patient-initiated ratings, n (%)	Surgeons with ≥10 total patient-initiated ratings, n (%)
All	23,171 (11.52)	10 (1-32)	26.37 (53.24)	189,463 (94.19)	158,630 (78.86)	103,600 (51.50)
Cardiothoracic (n=5505)	867 (15.75)	6 (0-16)	12.33 (22.60)	5094 (92.53)	4116 (74.77)	2184 (39.67)
Colorectal (n=1990)	535 (26.88)	20 (4-45)	32.97 (48.44)	1850 (92.96)	1683 (84.57)	1280 (64.32)
General (n=39,343)	3028 (7.70)	4 (0-16)	13.46 (31.18)	36,190 (91.99)	26,738 (67.96)	13,598 (34.56)
Neurosurgery (n=7574)	965 (12.74)	18 (2-51)	38.14 (64.10)	7110 (93.87)	6179 (81.58)	4571 (60.35)
OB/GYN^a^ (n=51,293)	3275 (6.38)	12 (2-34)	24.82 (39.64)	48,461 (94.48)	41,389 (80.69)	27,492 (53.60)
Ophthalmology (n=24,227)	3546 (14.64)	8 (1-24)	22.77 (63.30)	23,008 (94.97)	19,211 (79.30)	11,551 (47.68)
Orthopedic (n=33,611)	4396 (13.08)	21 (3-54)	42.91 (69.63)	31,986 (95.17)	28,237 (84.01)	21,500 (63.97)
Otolaryngology (n=13,394)	2549 (19.03)	13 (2-34)	28.92 (66.66)	12,710 (94.89)	10,912 (81.47)	7522 (56.16)
Plastic (n=7065)	1398 (19.79)	21 (4-50)	42.51 (77.86)	6686 (94.64)	6038 (85.46)	4617 (65.35)
Urology (n=13,552)	1982 (14.63)	14 (2-34)	25.35 (43.04)	12,930 (95.41)	11,243 (82.96)	7741 (57.12)
Vascular (n=3600)	630 (17.50)	7 (1-18)	15.12 (35.20)	3438 (95.50)	2884 (80.11)	1544 (42.89)

a OB/GYN: obstetrics and gynecology.

The number of ratings and overall ratings across different patient-initiated rating platforms is shown in Figure S1 in Multimedia Appendix 1. Across all specialties, a small number of surgeons received disproportionately high numbers of ratings, and the ratings were negatively skewed, with most aggregate ratings at around 4 out of 5, whereas relatively few were below 3 out of 5.

We identified 11.52% (23,171/201,154) of surgeons with “Top Doctor” status on the peer-nominated rating platform (Table 1). On the peer-nominated platform, the 3 surgical specialties with the highest proportion of surgeons recognized as “Top Doctors” were colorectal surgery (535/1990, 26.88%), plastic surgery (1398/7065, 19.79%), and otolaryngology (2549/13,394, 19.03%). The specialties with the lowest rates of recognition were obstetrics and gynecology (3275/51,293, 6.38%) and general surgery (3028/39,343, 7.70%; Table 1).

### Geographic Distribution of Surgeons on Online Rating Platforms

**Figure 2.**
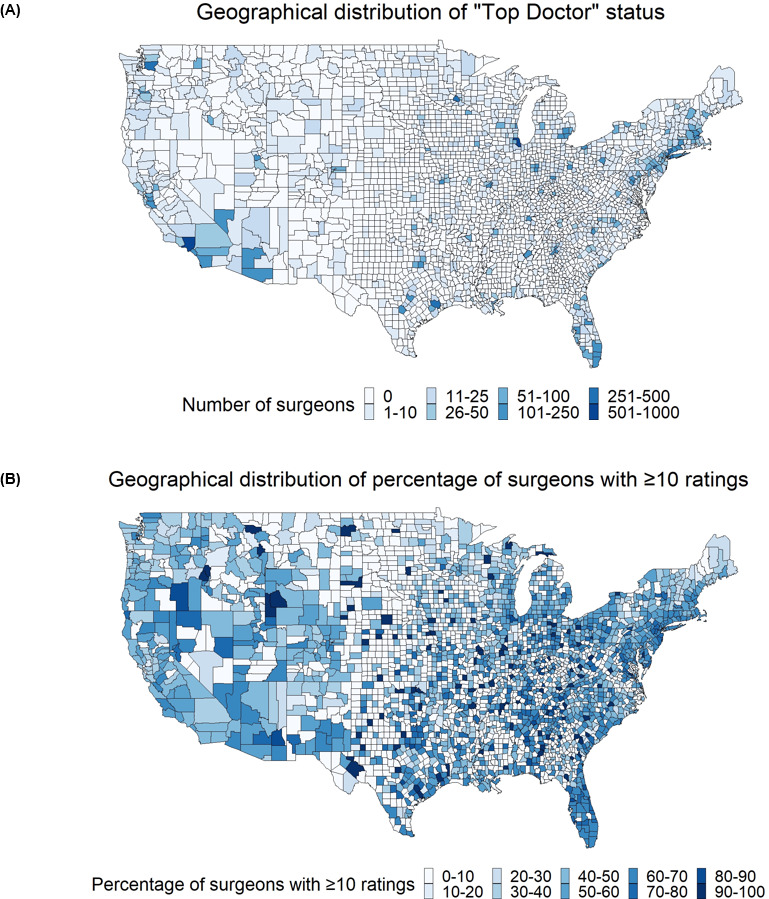
(A) Geographical distribution of Castle Connolly “Top Doctors” in the United States in this cross-sectional study. (B) Geographical distribution of surgeons with 10 or more ratings in the United States. We mapped the zip codes of each surgeon’s primary practice location to their local county to generate a county-specific number of “Top Doctor” surgeons and the percentage of surgeons with 10 or more ratings for the contiguous United States (197,019/201,154, 97.94% of the total directory). Counties that had 0 surgeons were assigned a percentage of 0.

The geographic distribution of surgeons with 10 or more patient-initiated ratings and “Top Doctor” status is shown in Figure 2. The practice locations of our surgeon cohort included 2199 US counties, which represents coverage for 94.9% of the US population (308,139,520 persons across all counties, with an estimated US population of 324,870,510) [9-11,13]. We found evidence of wide coverage across the United States of both the patient-initiated and peer-nominated platforms. A total of 85.18% (1873/2199) of the counties, covering 91.06% (295,816,471/324,870,510) of the population, had at least one surgeon with 10 or more ratings, and 48.93% (1076/2199) of the counties, covering 82.03% (266,475,236/324,870,510) of the population, had at least one surgeon with “Top Doctor” status (Figure 2). We found that Los Angeles, Cook, New York, Maricopa, Harris, Miami-Dade, Orange, San Diego, Nassau, and Dallas, in order from highest to lowest, were the counties with the highest number of surgeons with 10 or more patient-initiated ratings. We found a similar pattern of geographic distribution for surgeons recognized as “Top Doctors,” with a higher presence in the US northeast.

### Relationship Between Rating Volume and Overall Rating Across Surgical Specialties

We examined differences in rating volume, stratifying surgeons by specialty and rating classification (<4, 4-4.5, and ≥4.5 out of 5; Table S2 in Multimedia Appendix 1). Among surgeons in the lowest and highest rating groups, there was a higher concentration of profiles with a low number (<10) of ratings, suggesting insufficient sampling. However, we also found that surgeons with high numbers of ratings (>100) were more likely to be in the highest (≥4.5) rating group (Figure S2 in Multimedia Appendix 1). Cardiothoracic surgery, plastic surgery, general surgery, and ophthalmology were the specialties with the highest proportion of surgeons with ratings of 4.5 or higher (Table S2 in Multimedia Appendix 1).

**Figure 3.**
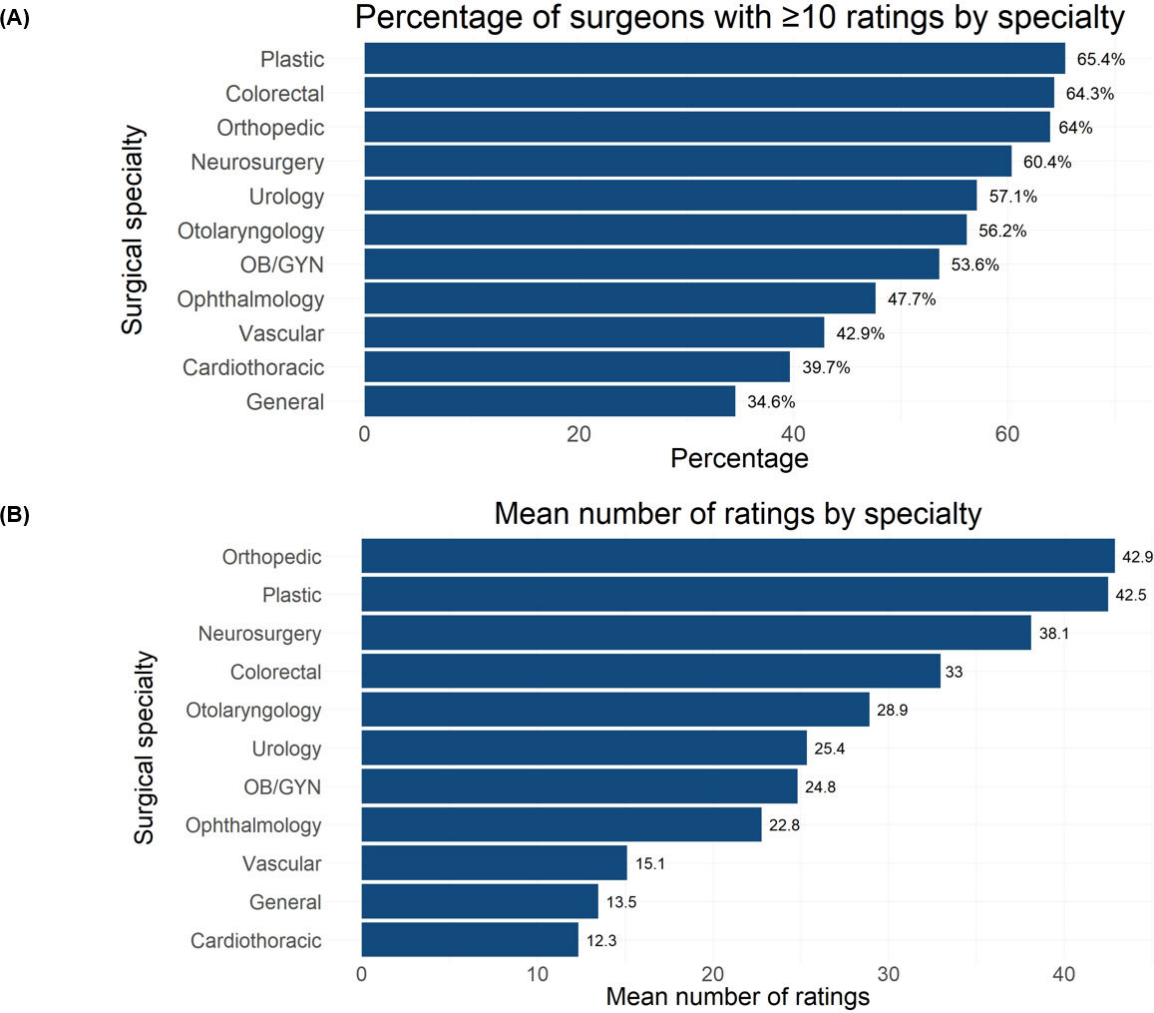
(A) Bar plot showing the percentage of surgeons in the United States with 10 or more ratings by each specialty in this cross-sectional study. (B) Bar plot showing the mean number of ratings for surgeons in the United States by each specialty in this cross-sectional study. OB/GYN: obstetrics and gynecology.

We identified substantial variation in the proportion of surgeons with 10 or more ratings across surgical specialties (Figure 3A). While specialties such as plastic surgery, colorectal surgery, orthopedic surgery, and neurosurgery had over 60% of surgeons with 10 or more ratings, specialties such as general surgery, cardiothoracic surgery, and vascular surgery had less than 45% of surgeons with 10 or more ratings. There was substantial variation in the mean number of ratings across specialties, with orthopedic and plastic surgery averaging over 40 ratings per surgeon, whereas general and cardiothoracic surgery averaged fewer than 15 ratings per surgeon (Figure 3B).

### Relationship Between Peer-Nominated and Patient-Initiated Platforms

**Figure 4.**
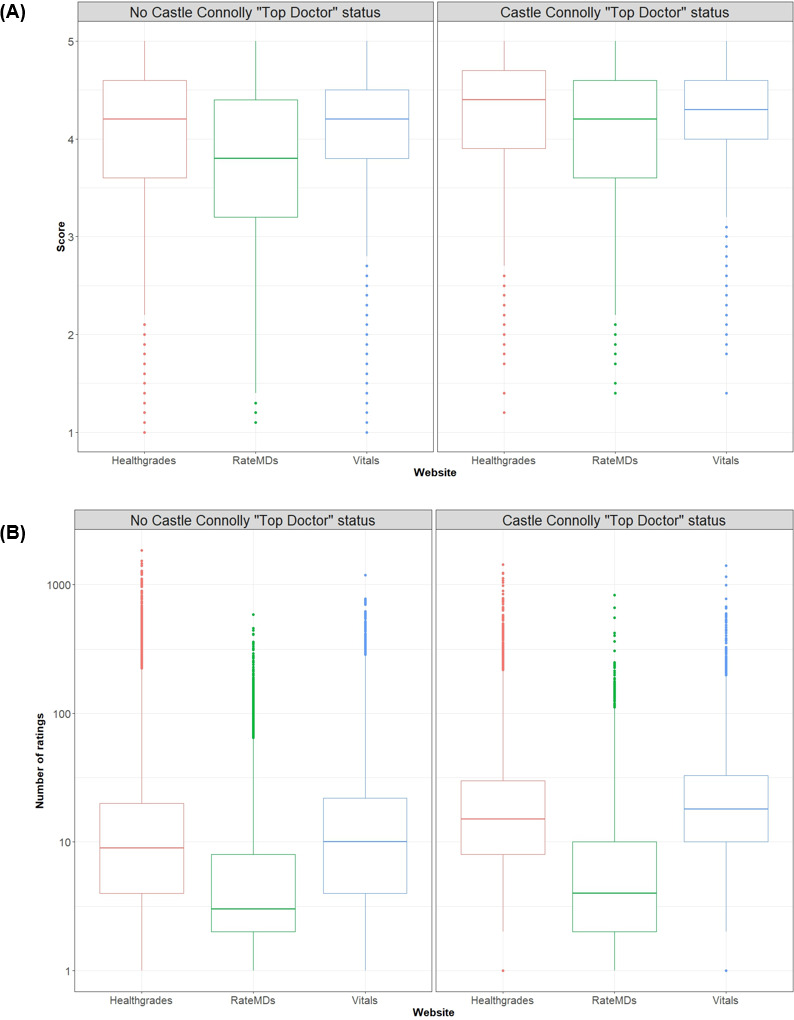
(A) Box plots of rating scores for surgeons in the United States with 10 or more ratings, stratified by peer-nominated “Top Doctor” status for each patient-initiated rating platform in this cross-sectional study. (B) Box plots of rating volume for surgeons in the United States with 10 or more ratings, stratified by peer-nominated “Top Doctor” status for each patient-initiated rating platform in this cross-sectional study.

We explored the relationship between peer-nominated and patient-initiated rating platforms and found that surgeons recognized as “Top Doctors” had higher median ratings across all patient-initiated rating platforms compared to their non–“Top Doctor” counterparts (Healthgrades: 4.36, IQR 3.88-4.71 vs 4.20, IQR 3.64-4.64, *P*<.001, and *r*=0.09; Vitals: 4.30, IQR 4.00-4.60 vs 4.20, IQR 3.80-4.50, *P*<.001, and *r*=0.09; RateMDs: 4.20, IQR 3.8-4.5 vs 3.80, IQR 3.60-4.60, *P*<.001, and *r*=0.16; Figure 4A). Additionally, surgeons recognized as “Top Doctors” had a greater median number of ratings across all platforms (Healthgrades: 15, IQR 7-29 vs 6, IQR 1-16, *P*<.001, and *r*=0.23; Vitals: 17, IQR 9-32 vs 7, IQR 1-18, *P*<.001, and *r*=0.23; RateMDs: 2, IQR 0-7 vs 1, IQR 0-4, *P*<.001, and *r*=0.12; Figure 4B).

### Characteristics of Surgeons With a High Number of Patient-Initiated Ratings

Finally, we explored specialty-specific patterns among surgeons with a high number of patient-initiated ratings. We found 133 individual surgeons with at least 500 ratings on 1 of the 3 platforms, with median ratings of 4.80 (IQR 4.70-4.90), 4.90 (IQR 4.90-5.00), and 4.85 (IQR 4.70-4.90) on Healthgrades, Vitals, and RateMDs, respectively. We also found considerable variation in the number of reviews required to be in the top 1 percentile across specialties, with cardiothoracic surgeons having 124 total ratings compared to plastic surgeons with 372 total ratings. At a specialty-specific level, surgeons in plastic surgery (372 ratings), orthopedic surgery (324 ratings), and neurosurgery (280 ratings) had the highest threshold for the number of ratings necessary to reach the top 1 percentile.

## Discussion

### Principal Findings

We found that 78.86% (158,630/201,154) of surgeons in the United States had at least one rating on a patient-initiated platform, and 11.52% (23,171/201,154) were recognized as “Top Doctors” by their peers. A total of 91.06% (295,816,471/324,870,510) of the population in the continental United States lives in a county with at least one surgeon with more than 10 reviews. Major metropolitan areas contained the highest frequency of surgeons with a high number of patient-initiated ratings, as well as those designated with a “Top Doctor” status. There was considerable variation in the number of highly rated surgeons on patient-initiated and peer-nominated platforms between specialties. Surgeons who had “Top Doctor” status were also more likely to receive a higher volume of ratings and overall ratings on patient-initiated rating platforms.

### Comparison With Prior Work

Previous studies have largely examined physician rating websites that are patient-initiated within specific specialties and/or regional locations [9,10,20-29]. Our study included both patient-initiated and peer-nominated rating platforms across all surgical subspecialties with comprehensive coverage of surgeons across the United States. Compared to a recent study that showed that 51.5% of physicians have at least one rating across 5 different patient-initiated rating platforms, we found meaningfully higher representation among surgeons on 3 different platforms that we identified as having the highest online traffic volume [17]. While there is inconsistent evidence regarding associations between surgeons being recognized by their peers as “Top Doctors” and both the number of patient-initiated ratings and overall patient ratings [14,27], we found that the median number of patient-initiated ratings and median ratings across all 3 patient-initiated rating platforms was statistically significantly higher (*P*<.001) among surgeons recognized as “Top Doctors.”

Our study focused specifically on the quantitative ratings on patient-initiated platforms. Prior studies of rating websites have noted that written reviews often describe characteristics aside from surgical outcomes, including bedside manner, wait times, communication with staff, and office environment [19,21,24,30-47]. We found that the rating scores of surgeons across all specialties were largely skewed toward a higher rating, and these findings suggest that patients use these platforms for positive feedback more commonly than for grievances, as feared by physicians. Prior studies have also reported similar findings based on quantitative and text analyses [48,49]. The extent to which these patient-initiated and peer-nominated websites capture patients’ experience compared to other review systems (eg, Press Ganey surveys) remains to be elucidated. Thematic or qualitative analyses of free-text patient comments (eg, sentiment analysis) may offer additional insights into patient considerations in rating surgeons.

### Limitations

Our study has limitations consistent with the study design. The rating websites used may have incorrect or outdated information and duplicate profiles. The data collected represent a snapshot of ratings, and changes over time to the ratings were not captured. Following the methods of Wang et al [17], much of the data were linked to the profiles found on Google search results and subsequently mapped to the name, specialty, and location in the surgeon directory. However, some ambiguities exist with common names or differences between nicknames found on online profiles and legal names used for official licensing. We programmatically resolved these by matching other factors such as location and specialty to further match profiles with different first names. The remaining cases that could not be programmatically resolved were addressed via manual review. Through automated Google searches to identify the profiles, there is the potential for profiles to be missing if they did not appear early (within the first 2 pages) in the search results. Google search results may return the profiles of related physicians in the same city or those with similar names. Our high rate of physician profile retrieval indicates that most profiles were captured, and subsequent data cleaning served to minimize errors.

### Mixed Incentives and Biases of Online Surgeon Rating Platforms

Our findings underscore the ubiquity and accessibility of online surgeon ratings, with top online search results of surgeons consistently yielding online profiles on several physician rating platforms. This widespread visibility highlights the growing influence of these ratings on patient perceptions and surgeon selection; however, notable disparities exist across surgical specialties and geographic regions on these online rating platforms. Patient-initiated online ratings are not regulated (eg, lack of patient verification) and, thus, may allow for exceedingly positive or negative reviews, which are not reflective of the direct patient-surgeon experience [30-32,50]. However, we found in this study that most surgeons had aggregate ratings at 4 out of 5 stars or higher, suggesting that ratings are typically positive overall and concerns that rating platforms display disproportionately negative experiences may be unfounded. While these rating websites aim to provide reputable assessment of surgeons by physician peers and patients, the accuracy of these platforms may instead be affected by financial incentives to promote a favorable online presence. Some of these rating platforms sell marketing collateral and promotional products that are eligible for highly rated physicians. It is also possible that soliciting reviews from patients may lead to biased results; consistent with this, we observed that the cohort of 133 surgeons with 500 or more patient reviews on a single platform often had near-perfect ratings. Future efforts to improve the bias in these rating systems are needed, and comparison of the ratings on these online platforms to independent third-party surveys from verified patients (eg, the Hospital Consumer Assessment of Healthcare Providers and Systems by the CMS) may be warranted.

### Conclusions

Surgeons across all specialties and locations are well represented on online rating platforms, with considerable variation in the volume of ratings between individual surgeons, across surgical specialties, and across a wide geographic distribution. The prevalence of these rating platforms justifies future work to understand the relationship between these ratings and how patients choose surgeons, as well as the association between ratings and objective measures of surgical outcomes.

## Supplementary material

10.2196/79427 Multimedia Appendix 1 Additional information across surgical specialties and rating platforms.

## Abbreviations

CMS: Centers for Medicare and Medicaid Services
STROBE: Strengthening the Reporting of Observational Studies in Epidemiology
