# Supplementary material for: Coverage, Traits, and Geographic Distribution of Online Surgeon Reviews: Large-Scale Cross-Sectional Analysis
Source: JMIR Form Res. 2026 Apr 14;10:e79427. doi: 10.2196/79427 (PMC13078610; doi:10.2196/79427)
Supplement: Multimedia Appendix 1 [file formative-v10-e79427-s001.docx]

Table S1. Table showing number of ratings and weighted averages across specialties

| Specialty | Total Number of Ratings, median (IQR) | Total Number of Ratings, mean (SD) | Weighted Average, median (IQR) | Weighted Average, mean (SD) |
| --- | --- | --- | --- | --- |
| Total | 10 (31) | 26.37  (53.24) | 4.26 (0.99) | 4.12 (0.78) |
| Cardiothoracic | 6 (16) | 12.33 (22.6) | 4.52 (0.92) | 4.37 (0.71) |
| Colorectal | 20 (41) | 32.97 (48.44) | 4.3 (0.76) | 4.21 (0.63) |
| General | 4 (16) | 13.46 (31.48) | 4.4 (1.04) | 4.21 (0.83) |
| Neurosurgery | 18 (49) | 38.14,  (64.1) | 4.21 (0.91) | 4.13 (0.7) |
| OB/GYN | 12 (32) | 24.82.  (39.64) | 4.18 (0.95) | 4.04 (0.79) |
| Ophthalmology | 8 (23) | 22.77 (63.3) | 4.33 (0.96) | 4.19 (0.75) |
| Orthopedic | 21 (51) | 42.91  (69.63) | 4.27 (0.91) | 4.13 (0.73) |
| Otolaryngology | 13 (32) | 28.92  (66.66) | 4.13 (1.03) | 4.02 (0.78) |
| Plastic | 21 (46) | 42.51  (77.86) | 4.34 (0.79) | 4.23 (0.66) |
| Urology | 14 (32) | 25.35  (43.04) | 4.02 (1.00) | 3.94 (0.79) |
| Vascular | 7 (17) | 15.12  (35.2) | 4.29 (1.05) | 4.15 (0.80) |

Table S2. Table showing top 25 counties by number of surgeons with ≥10 ratings

| Rank | Count | County | State | Population |
| --- | --- | --- | --- | --- |
| 1 | 2950 | Los Angeles | California | 10170292 |
| 2 | 2073 | Cook | Illinois | 5238216 |
| 3 | 2060 | New York | New York | 1644518 |
| 4 | 1516 | Maricopa | Arizona | 4167947 |
| 5 | 1443 | Harris | Texas | 4538028 |
| 6 | 1098 | Miami-Dade | Florida | 2693117 |
| 7 | 1092 | Orange | California | 3169776 |
| 8 | 1002 | San Diego | California | 3299521 |
| 9 | 999 | Nassau | New York | 1361350 |
| 10 | 920 | Dallas | Texas | 2553385 |
| 11 | 851 | King | Washington | 2117125 |
| 12 | 776 | Broward | Florida | 1896425 |
| 13 | 732 | Oakland | Michigan | 1242304 |
| 14 | 724 | Cuyahoga | Ohio | 1255921 |
| 15 | 691 | Palm Beach | Florida | 1422789 |
| 16 | 680 | Hillsborough | Florida | 1349050 |
| 17 | 668 | Bexar | Texas | 1897753 |
| 18 | 630 | Suffolk | New York | 1501587 |
| 19 | 622 | Philadelphia | Pennsylvania | 1567442 |
| 20 | 612 | Fulton | Georgia | 1010562 |
| 21 | 612 | Allegheny | Pennsylvania | 1230459 |
| 22 | 609 | Tarrant | Texas | 1982498 |
| 23 | 602 | Suffolk | Massachusetts | 778121 |
| 24 | 600 | Bergen | New Jersey | 938506 |
| 25 | 593 | Orange | Florida | 1288126 |

Table S3. Table showing top 25 counties by number of surgeons with “Top Doctor” recognition

| Rank | Surgeon Count | County | State | Population |
| --- | --- | --- | --- | --- |
| 1 | 951 | New York | New York | 1644518 |
| 2 | 597 | Los Angeles | California | 10170292 |
| 3 | 540 | Cook | Illinois | 5238216 |
| 4 | 356 | Harris | Texas | 4538028 |
| 5 | 345 | King | Washington | 2117125 |
| 6 | 329 | Suffolk | Massachusetts | 778121 |
| 7 | 326 | Nassau | New York | 1361350 |
| 8 | 285 | Westchester | New York | 976396 |
| 9 | 283 | Philadelphia | Pennsylvania | 1567442 |
| 10 | 269 | Hennepin | Minnesota | 1223149 |
| 11 | 250 | Orange | California | 3169776 |
| 12 | 244 | San Diego | California | 3299521 |
| 13 | 233 | Cuyahoga | Ohio | 1255921 |
| 14 | 230 | Allegheny | Pennsylvania | 1230459 |
| 15 | 229 | Miami-Dade | Florida | 2693117 |
| 16 | 218 | Bergen | New Jersey | 938506 |
| 17 | 218 | Milwaukee | Wisconsin | 957735 |
| 18 | 217 | Maricopa | Arizona | 4167947 |
| 19 | 212 | Fairfield | Connecticut | 948053 |
| 20 | 209 | San Francisco | California | 864816 |
| 21 | 201 | Franklin | Ohio | 1251722 |
| 22 | 200 | Fulton | Georgia | 1010562 |
| 23 | 189 | Mecklenburg | North Carolina | 1034070 |
| 24 | 188 | Travis | Texas | 1176558 |
| 25 | 187 | Santa Clara | California | 1918044 |

Table S4. Rating counts by rating categories (<4, 4-4.5, ≥4.5) and by specialty, including only surgeons with ≥10 ratings

| **Specialty** | **Surgeons, n** | **<4, n (%)** | **4-4.50, n (%)** | **≥4.5, n (%)** |
| --- | --- | --- | --- | --- |
| Cardiothoracic | 2184 | 408 (18.7) | 769 (35.2) | 1007 (46.1) |
| Plastic | 4617 | 1301 (28.2) | 1663 (36.0) | 1653 (35.8) |
| General | 13599 | 3888 (28.6) | 4876 (35.9) | 4835 (35.6) |
| Colorectal | 1280 | 378 (29.5) | 525 (41.0) | 377 (29.5) |
| Ophthalmology | 11551 | 3758 (32.5) | 3841 (33.3) | 3952 (34.2) |
| Orthopedic | 21500 | 7412 (34.5) | 7103 (33.0) | 6985 (32.5) |
| Vascular | 1544 | 537 (34.8) | 540 (35.0) | 467 (30.2) |
| Neurosurgery | 4571 | 1776 (38.9) | 1612 (35.3) | 1183 (25.9) |
| OB/GYN | 27492 | 11278 (41.0) | 9520 (34.6) | 6694 (24.3) |
| Otolaryngology | 7522 | 3400 (45.2) | 2281 (30.3) | 1841 (24.5) |
| Urology | 7741 | 3929 (50.8) | 2381 (30.8) | 1431 (18.5) |
| Total | 103601 | 38065 (29.7) | 35111 (33.9) | 30425 (29.4) |

Table S5. Number of ratings required to meet top percentile thresholds across specialties.

| specialty | Top 1% | Top 5% | Top 10% | Top 25% | Top 50% |
| --- | --- | --- | --- | --- | --- |
| Cardiothoracic | 90 | 44 | 30 | 16 | 6 |
| Colorectal | 184 | 102 | 77 | 45 | 20 |
| General | 124 | 54 | 35 | 16 | 4 |
| Neurosurgery | 280 | 137 | 95 | 51 | 18 |
| OB/GYN | 166 | 89 | 64 | 34 | 12 |
| Ophthalmology | 225 | 78 | 50 | 24 | 8 |
| Orthopedic | 324 | 162 | 108 | 54 | 21 |
| Otolaryngology | 261 | 96 | 64 | 34 | 13 |
| Plastic | 372 | 148 | 99 | 50 | 21 |
| Urology | 180 | 84 | 61 | 34 | 14 |
| Vascular | 116 | 48 | 33 | 18 | 7 |

Table S6. Number of ratings required to meet top percentile thresholds across specialties for Healthgrades.

| Specialty | Top 1% | Top 5% | Top 10% | Top 25% | Top 50% |
| --- | --- | --- | --- | --- | --- |
| Cardiothoracic | 49 | 21 | 14 | 7 | 1 |
| Colorectal | 102 | 48 | 35 | 20 | 8 |
| General | 67 | 25 | 16 | 7 | 1 |
| Neurosurgery | 154 | 65 | 43 | 22 | 6 |
| OB/GYN | 84 | 38 | 26 | 14 | 4 |
| Ophthalmology | 143 | 43 | 24 | 10 | 3 |
| Orthopedic | 197 | 94 | 58 | 25 | 9 |
| Otolaryngology | 138 | 48 | 31 | 16 | 5 |
| Plastic | 165 | 59 | 39 | 19 | 7 |
| Urology | 93 | 41 | 28 | 15 | 5 |
| Vascular | 72 | 24 | 15 | 8 | 3 |

Table S7. Number of ratings required to meet top percentile thresholds across specialties for Vitals.

| specialty | Top 1% | Top 5% | Top 10% | Top 25% | Top 50% |
| --- | --- | --- | --- | --- | --- |
| Cardiothoracic | 9 | 3 | 1 | 0 | 0 |
| Colorectal | 22 | 8 | 5 | 1 | 0 |
| General | 16 | 4 | 2 | 0 | 0 |
| Neurosurgery | 47 | 18 | 11 | 3 | 0 |
| OB/GYN | 32 | 16 | 9 | 2 | 0 |
| Ophthalmology | 25 | 5 | 3 | 0 | 0 |
| Orthopedic | 42 | 14 | 8 | 2 | 0 |
| Otolaryngology | 43 | 14 | 8 | 2 | 0 |
| Plastic | 80 | 30 | 16 | 4 | 0 |
| Urology | 26 | 9 | 5 | 1 | 0 |
| Vascular | 7 | 3 | 1 | 0 | 0 |

Table S8. Number of ratings required to meet top percentile thresholds across specialties for RateMDs.

| specialty | Top 1% | Top 5% | Top 10% | Top 25% | Top 50% |
| --- | --- | --- | --- | --- | --- |
| Cardiothoracic | 45 | 25 | 17 | 9 | 2 |
| Colorectal | 94 | 56 | 44 | 25 | 8 |
| General | 58 | 27 | 18 | 8 | 1 |
| Neurosurgery | 117 | 64 | 46 | 25 | 6 |
| OB/GYN | 82 | 45 | 32 | 17 | 4 |
| Ophthalmology | 75 | 34 | 23 | 12 | 3 |
| Orthopedic | 136 | 66 | 46 | 24 | 8 |
| Otolaryngology | 97 | 41 | 29 | 15 | 4 |
| Plastic | 168 | 75 | 50 | 25 | 8 |
| Urology | 76 | 41 | 30 | 17 | 6 |
| Vascular | 54 | 26 | 18 | 10 | 2 |

Figure S1A. Boxplot of the number of ratings across surgical specialties.

Figure S1B. Boxplot of the scores across surgical specialties (only for surgeons with ≥10 ratings)


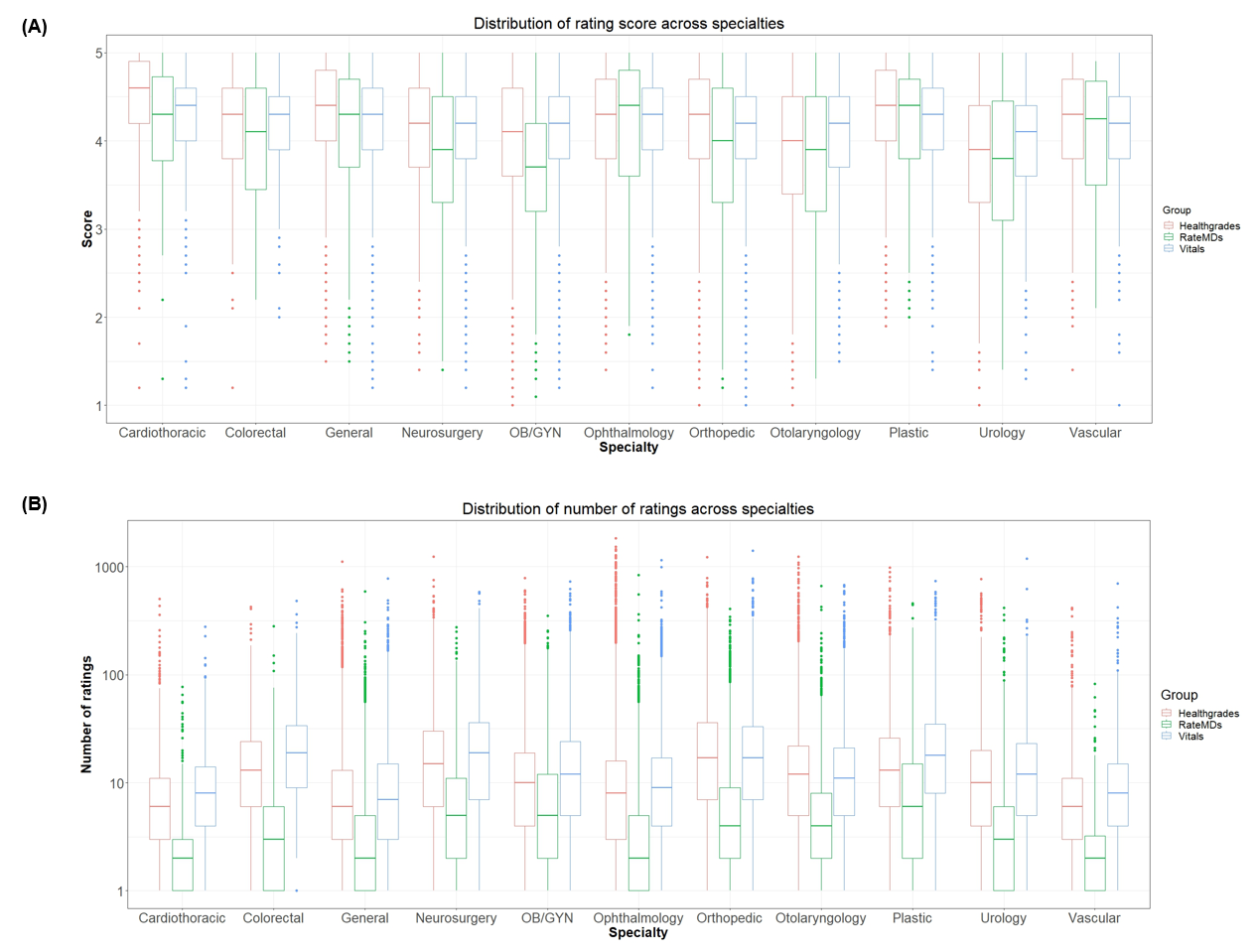


Figure S2. Density Plot of Total Ratings Across All Rating Groups (<4.0, 4.0-4.5, and ≥4.5)


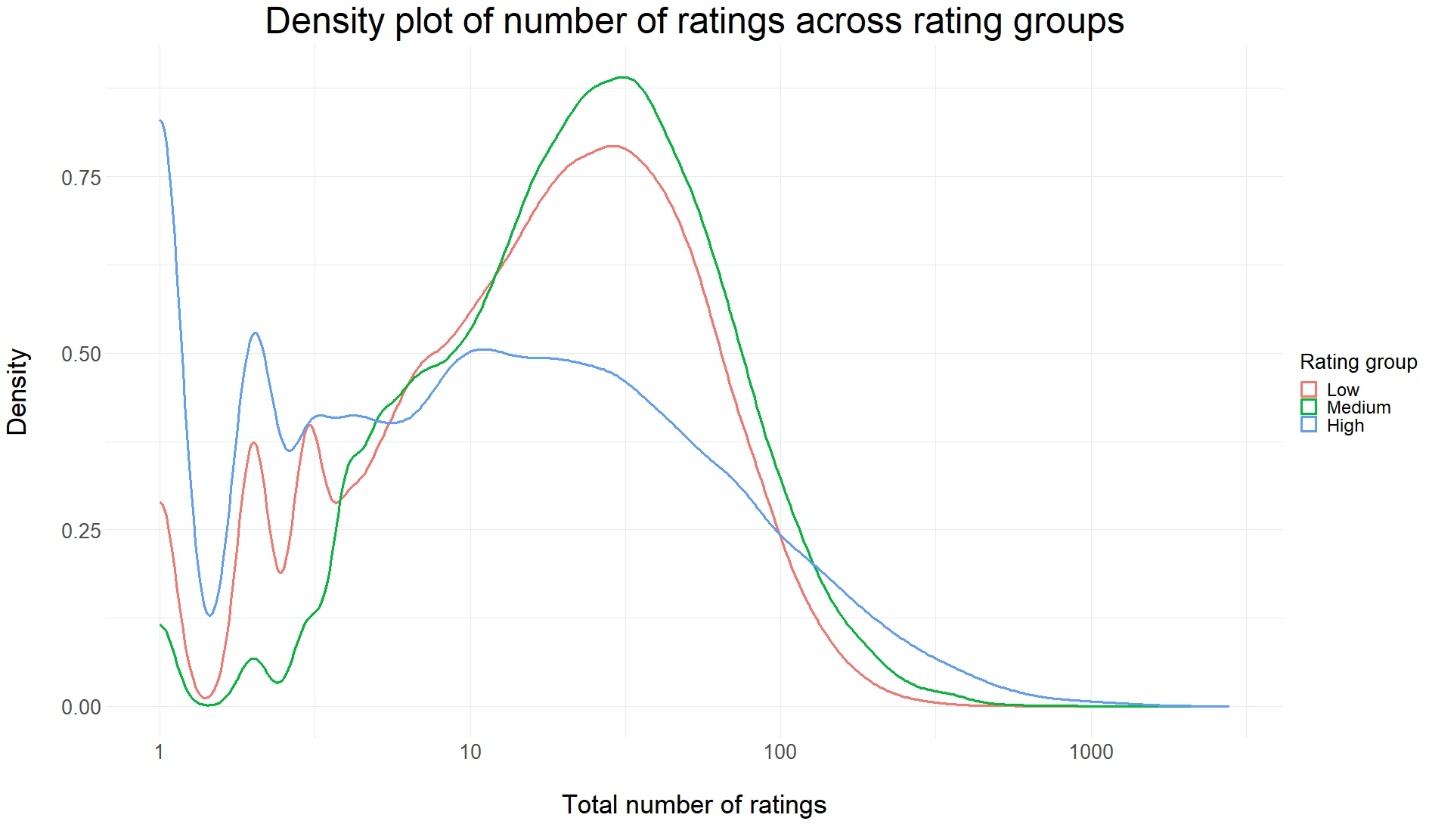


Figure S3. Specialty Representation Among Surgeons with ≥10 Ratings Stratified by Rating Group (<4.0, 4.0-4.50, and ≥4.5)
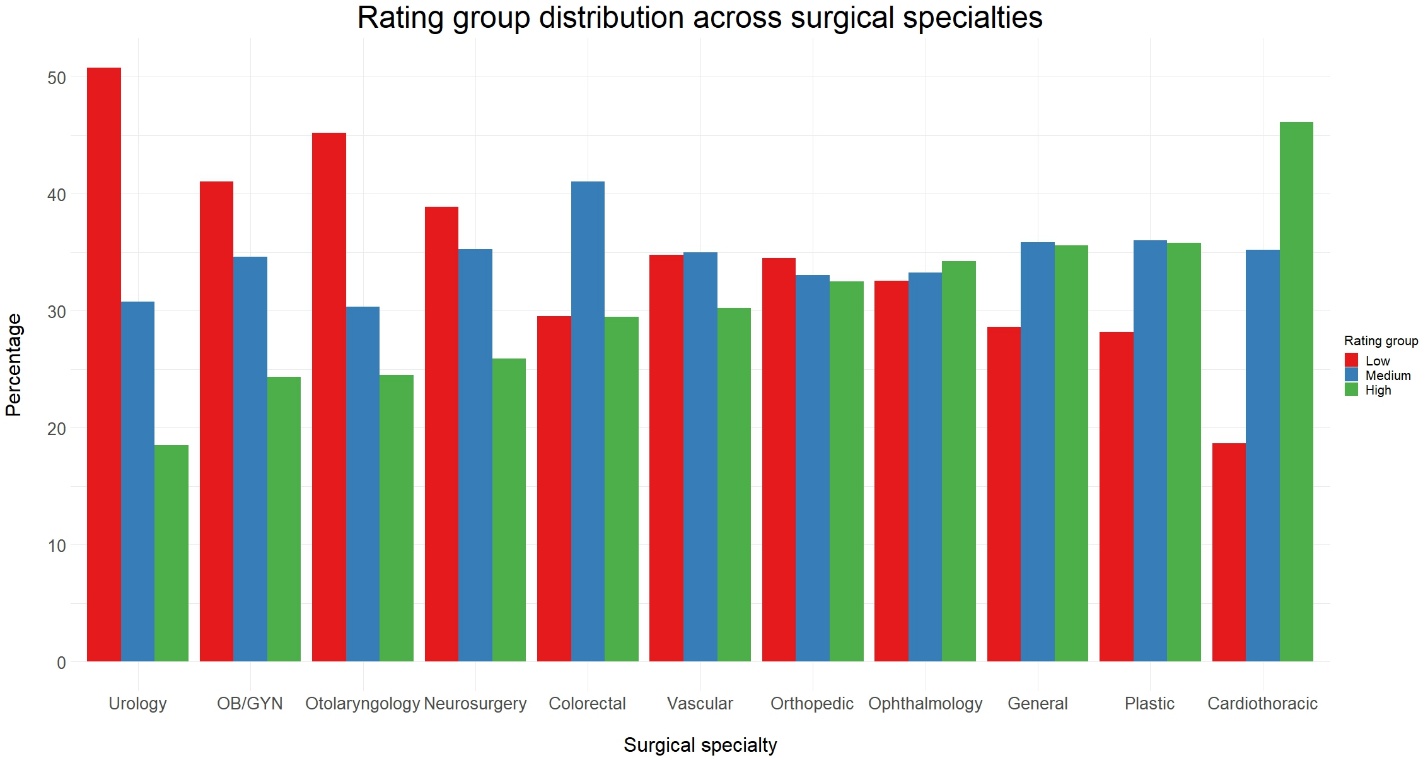


Figure S4. Specialty Overrepresentation in the Most Rated Surgeon Profiles.


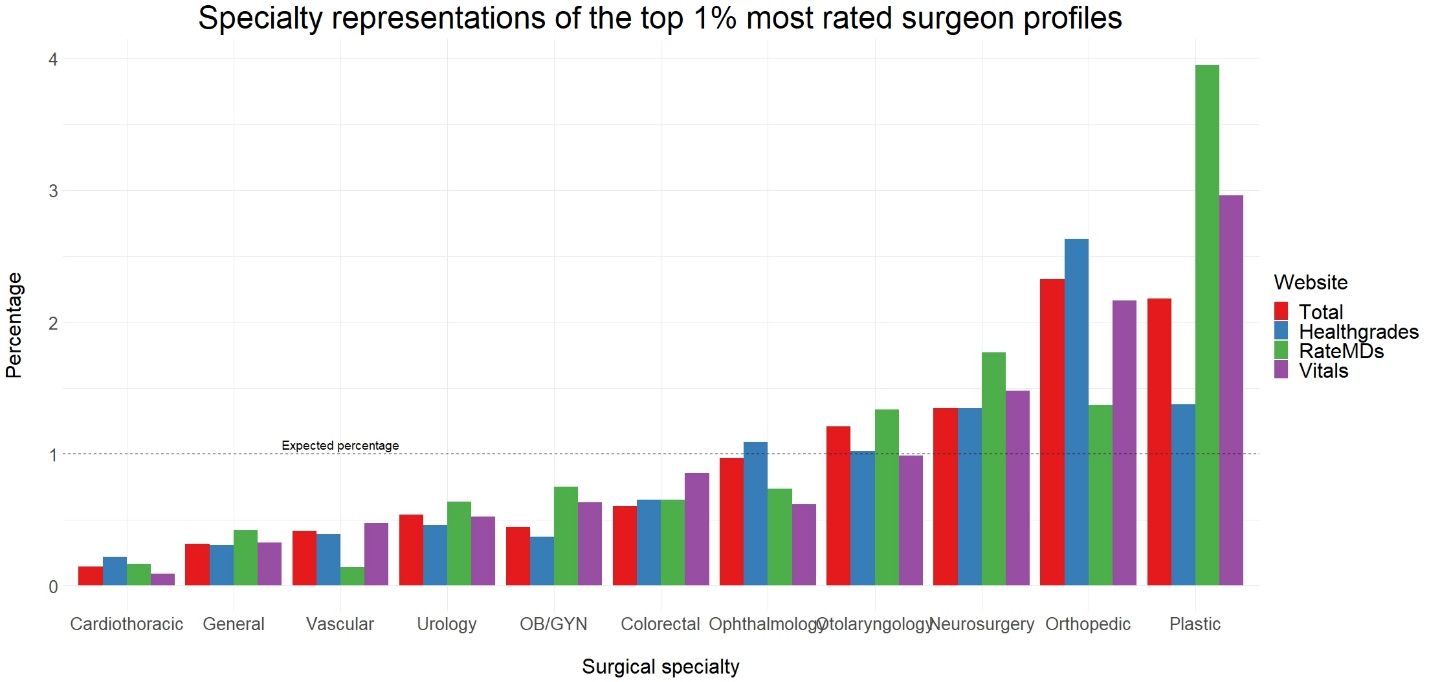


Figure S4 Legend. Among the top 1% of most rated profiles, we plotted the percentage of surgeons within each individual specialty instead of the counts to normalize surgeon population differences across specialties. If every field were uniformly represented when selecting the top 1% of overall surgeons, then 1% of each surgeon population by specialty is expected.
